# Supplementary material for: Efficacy and safety of non-pharmacological therapy under the guidance of TCM theory in the treatment of anxiety in patients with myocardial infarction: A protocol for systematic review and meta-analysis
Source: PLoS One. 2023 Jul 6;18(7):e0288154. doi: 10.1371/journal.pone.0288154 (PMC10325115; doi:10.1371/journal.pone.0288154)
Supplement: S1 File — (DOC) [file pone.0288154.s001.doc]

# Search strategy

**PubMed**

#1 Miocardial infarction [mh] OR Miocardial infarction [tiab] OR Disease*, Infarction, Myocardial [tiab] OR Infarction, Myocardial[tiab] OR Myocardial Infarctions[tiab] OR Cardiovascular Stroke[tiab] OR Cardiovascular Strokes[tiab] OR Stroke, Cardiovascular[tiab] OR Myocardial Infarct[tiab] OR Infarct, Myocardial[tiab] OR Angina, Unstable[mh] OR Anginas, Unstable[tiab] OR Unstable Anginas[tiab] OR Angina Pectoris, Unstable[tiab] OR Angina Pectori, Unstable[tiab] OR Unstable Angina Pectori[tiab] OR Unstable Angina[tiab] OR Angina at Rest[tiab] OR Angina, Preinfarction[tiab] OR Preinfarction Angina[tiab] OR Preinfarction Anginas[tiab] OR Myocardial Preinfarction Syndrome[tiab] OR Myocardial Preinfarction Syndromes[tiab] OR Preinfarction Syndrome, Myocardial[tiab] OR Syndrome, Myocardial Preinfarction[tiab] OR Chronic Stable Angina[tiab] OR Angina Pectoris, Stable[tiab] OR Stable angina[tiab] OR Syndromes, Myocardial Preinfarction[tiab] OR percutaneous coronary intervention[tiab] OR PCI[tiab] OR coronary artery bypass grafting[tiab]

#2 Anxiety[mh] OR Anxiety[tiab] OR Social Anxiet*[tiab] OR Anxiet*, Social[tiab] OR Hypervigilance[tiab] OR Nervousness[tiab] OR Anxiousness[tiab] OR Performance Anxiety[mh] OR Anxieties, Performance[tiab] OR Anxiety, Performance[tiab] OR Performance Anxieties[tiab] OR panic [tiab] OR obsessive compulsive disorder [tiab] OR generalized anxiety disorder [tiab] OR GAD[tiab] OR agoraphobia[tiab] OR phobic disorders[tiab] OR stress disorders[tiab] OR post-traumatic stress disorder[tiab] OR PTSD[tiab]

#3 #1 AND #2

#4 Complementary Therapies [mh] OR Complementary [tiab] OR Therapies, Complementary [tiab] herapy, Complementary [tiab] OR Alternative Medicine [tiab] OR Therapies, Alternative[tiab] OR Therapy, Alternative [tiab] OR Non-pharmacological therapy [mh] OR Nonpharmacological therapy [mh] OR Non-pharmacological therapy [tiab] OR Nonpharmacological therapy [tiab]

#5 Acupuncture [mh] OR Acupuncture* [tiab] OR Acupuncture [tiab] OR Massage [mh] OR Massage* [tiab] OR massage [tiab] OR acupoint [tiab] OR Traditional Chinese Medicine Utilitarian [tiab] OR utilitarian [tiab] OR Taiji [mh] OR Taiji[tiab] OR "Tai Chi"[tiab] OR "Tai-Ji"[tiab] OR "Tai-Chi"[tiab] OR Qigong [mh] OR Qigong[tiab] OR Wuqinxi [tiab] OR Tendon Changing Classic [tiab] OR Yijinjing[tiab]

#6 #4 OR #5

#7 Randomized controlled trial[pt] OR controlled clinical trial[pt] OR randomized[tiab] OR placebo[tiab] OR randomly[tiab] OR trial[tiab] OR groups[tiab]

#8 meta analysis[tiab] OR cochrane review[tiab] OR systematic review[tiab]

#9 #7 OR #8

#10 #3 AND #6 AND #9

**COCHRANE**

#1 (Miocardial infarction OR Disease*, Infarction, Myocardial OR Infarction, Myocardial OR Myocardial Infarctions OR Cardiovascular Stroke OR Cardiovascular Strokes OR Stroke, Cardiovascular OR Myocardial Infarct OR Infarct, Myocardial OR Anginas, Unstable OR Unstable Anginas OR Angina Pectoris, Unstable OR Angina Pectori, Unstable OR Unstable Angina Pectori OR Unstable Angina OR Angina at Rest OR Angina, Preinfarction OR Preinfarction Angina OR Preinfarction Anginas OR Myocardial Preinfarction Syndrome OR Myocardial Preinfarction Syndromes OR Preinfarction Syndrome, Myocardial OR Syndrome, Myocardial Preinfarction OR Chronic Stable Angina OR Angina Pectoris, Stable OR Stable angina OR Syndromes, Myocardial Preinfarction OR percutaneous coronary intervention OR PCI OR coronary artery bypass grafting):ti,ab,kw

#2 MeSH descriptor: [Miocardial infarction] explode all trees

#3 MeSH descriptor: [Angina, Unstable] explode all trees

#4 #1 and#2 and#3

#5(Anxiety OR Social Anxiet* OR Anxiet*, Social OR Hypervigilance OR Nervousness OR Anxiousness OR Anxieties, Performance OR Anxiety, Performance OR Performance Anxieties OR panic OR obsessive compulsive disorder OR generalized anxiety disorder OR GAD OR agoraphobia OR phobic disorders OR stress disorders OR post-traumatic stress disorder OR PTSD):ti,ab,kw

#6 MeSH descriptor: [Anxiety] explode all trees

#7 MeSH descriptor: [Performance Anxiety] explode all trees

#8 #5 and#6 and#7

#9 (Complementary OR Complementary Therapies OR Therapies, Complementary herapy, Complementary OR Alternative Medicine OR Therapies, Alternative OR Therapy, Alternative OR Non-pharmacological therapy OR Nonpharmacological therapy OR Acupuncture* OR Acupuncture OR Massage* OR massage OR acupoint OR Traditional Chinese Medicine Utilitarian OR utilitarian OR Taiji OR "Tai Chi" OR "Tai-Ji" OR "Tai-Chi" OR Qigong OR Wuqinxi OR Tendon Changing Classic OR Yijinjing ):ti,ab,kw

#10 MeSH descriptor: [Complementary Therapies] explode all trees

#11 MeSH descriptor: [Non-pharmacological therapy] explode all trees

#12 MeSH descriptor: [Acupuncture] explode all trees

#13 MeSH descriptor: [massage] explode all trees

#14 MeSH descriptor: [Taiji] explode all trees

#15 MeSH descriptor: [Qigong] explode all trees

#16 MeSH descriptor: [Taiji] explode all trees

#17 #9 and#10 and#11 and#12 and#13 and#14 and#15 and#16

#18 (Randomized controlled trial OR controlled clinical trial OR randomized OR placebo OR randomly OR trial OR groups):ti,ab,kw

#19 (meta analysis OR cochrane review OR systematic review):ti,ab,kw

#20 #4 AND #8 AND #17 AND #18 AND #19

**Scopus**

TITLE-ABS-KEY (“Miocardial infarction*” OR “Miocardial infarction” OR “Disease*, Infarction, Myocardial” OR “Infarction, Myocardial” OR “Myocardial Infarctions” OR “Cardiovascular Stroke” OR “Cardiovascular Strokes” OR “Stroke, Cardiovascular” OR “Myocardial Infarct” OR “Infarct, Myocardial” OR “Angina, Unstable” OR “Anginas, Unstable” OR “Unstable Anginas” OR “Angina Pectoris, Unstable” OR “Angina Pectori, Unstable” OR “Unstable Angina Pectori” OR “Unstable Angina” OR “Angina at Rest” OR “Angina, Preinfarction” OR “Preinfarction Angina” OR “Preinfarction Anginas” OR “Myocardial Preinfarction Syndrome” OR “Myocardial Preinfarction Syndromes” OR “Preinfarction Syndrome, Myocardial” OR “Syndrome, Myocardial Preinfarction” OR “Chronic Stable Angina” OR “Angina Pectoris, Stable” OR “Stable angina” OR “Syndromes, Myocardial Preinfarction” OR “percutaneous coronary intervention” OR “PCI” OR “coronary artery bypass grafting”) AND TITLE-ABS-KEY (“Anxiety*” OR “Anxiety” OR “Social Anxiet*” OR “Anxiet*, Social” OR “Hypervigilance” OR “Nervousness” OR “Anxiousness” OR “Anxiety Performance” OR “Anxieties, Performance” OR “Anxiety, Performance” OR “Performance Anxieties” OR "panic" OR "obsessive compulsive disorder" OR "OCD" OR "generalized anxiety disorder" OR "GAD" OR "agoraphobia" OR "phobic disorders" OR "stress disorders" OR "post-traumatic stress disorder ") AND TITLE-ABS-KEY (“Complementary Therapies” OR “Complementary” OR “Therapies, Complementary herapy, Complementary” OR “Alternative Medicine” OR “Therapies, Alternative” OR “Therapy, Alternative “ OR “Non-pharmacological therapy” OR “Nonpharmacological therapy” OR “Non-pharmacological therapy” OR “Nonpharmacological therapy” OR “Acupuncture*” OR “Acupuncture” OR “Massage*” OR “massage” OR “acupoint” OR “Traditional Chinese Medicine Utilitarian” OR “utilitarian” OR “Taiji*” OR “Taiji” OR “"Tai Chi"“ OR “"Tai-Ji"“ OR “"Tai-Chi"“ OR “Qigong*” OR “Qigong” OR “Wuqinxi” OR “Tendon Changing Classic” OR “Yijinjing” ) AND TITLE-ABS-KEY ( “Randomized controlled trial*” OR “controlled clinical trial*” OR “randomized*” OR “placebo*” OR “randomly*” OR “trial*” OR “groups*” OR “meta analysis*” OR “cochrane review*” OR “systematic review*” )

**Ovid**

1# (Miocardial infarction* OR Miocardial infarction OR Disease*, Infarction, Myocardial OR Infarction, Myocardial OR Myocardial Infarctions OR Cardiovascular Stroke OR Cardiovascular Strokes OR Stroke, Cardiovascular OR Myocardial Infarct OR Infarct, Myocardial OR Angina, Unstable OR Anginas, Unstable OR Unstable Anginas OR Angina Pectoris, Unstable OR Angina Pectori, Unstable OR Unstable Angina Pectori OR Unstable Angina OR Angina at Rest OR Angina, Preinfarction OR Preinfarction Angina OR Preinfarction Anginas OR Myocardial Preinfarction Syndrome OR Myocardial Preinfarction Syndromes OR Preinfarction Syndrome, Myocardial OR Syndrome, Myocardial Preinfarction OR Chronic Stable Angina OR Angina Pectoris, Stable OR Stable angina OR Syndromes, Myocardial Preinfarction OR percutaneous coronary intervention OR PCI OR coronary artery bypass grafting).ti, ab.

2# (Anxiety* OR Anxiety OR Social Anxiet* OR Anxiet*, Social OR Hypervigilance OR Nervousness OR Anxiousness OR Anxiety Performance OR Anxieties, Performance OR Anxiety, Performance OR Performance Anxieties OR panic OR obsessive compulsive disorder OR generalized anxiety disorder OR GAD OR agoraphobia OR phobic disorders OR stress disorders OR post-traumatic stress disorder OR PTSD).ti, ab.

3# 1# AND 2#

4# **(**Complementary Therapies OR Complementary OR Therapies, Complementary herapy, Complementary OR Alternative Medicine OR Therapies, Alternative OR Therapy, Alternative OR Non-pharmacological therapy OR Nonpharmacological therapy OR Non-pharmacological therapy OR Nonpharmacological therapy).ti, ab.

5# **(**Acupuncture* OR Acupuncture OR Massage* OR massage OR acupoint OR Traditional Chinese Medicine Utilitarian OR utilitarian OR Taiji* OR Taiji OR "Tai Chi" OR "Tai-Ji" OR "Tai-Chi" OR Qigong* OR Qigong OR Wuqinxi OR Tendon Changing Classic OR Yijinjing).ti, ab.

6# (randomized controlled trial or controlled clinical trial).pt,mt. or randomized.ab. or placebo.ab. or randomly.ab. or trial.ab. or groups.ab.

7# 1#and4# and5# and6#

**Web of science**

1# TS=(Miocardial infarction* OR Miocardial infarction OR Disease*, Infarction, Myocardial OR Infarction, Myocardial OR Myocardial Infarctions OR Cardiovascular Stroke OR Cardiovascular Strokes OR Stroke, Cardiovascular OR Myocardial Infarct OR Infarct, Myocardial OR Angina, Unstable OR Anginas, Unstable OR Unstable Anginas OR Angina Pectoris, Unstable OR Angina Pectori, Unstable OR Unstable Angina Pectori OR Unstable Angina OR Angina at Rest OR Angina, Preinfarction OR Preinfarction Angina OR Preinfarction Anginas OR Myocardial Preinfarction Syndrome OR Myocardial Preinfarction Syndromes OR Preinfarction Syndrome, Myocardial OR Syndrome, Myocardial Preinfarction OR Chronic Stable Angina OR Angina Pectoris, Stable OR Stable angina OR Syndromes, Myocardial Preinfarction OR percutaneous coronary intervention OR PCI OR coronary artery bypass grafting)

2# TS=(Anxiety* OR Anxiety OR Social Anxiet* OR Anxiet*, Social OR Hypervigilance OR Nervousness OR Anxiousness OR Anxiety Performance OR Anxieties, Performance OR Anxiety, Performance OR Performance Anxieties OR panic OR obsessive compulsive disorder OR generalized anxiety disorder OR GAD OR agoraphobia OR phobic disorders OR stress disorders OR post-traumatic stress disorder OR PTSD)

3# 1# AND 2#

4# TS=(Complementary Therapies OR Complementary OR Therapies, Complementary herapy, Complementary OR Alternative Medicine OR Therapies, Alternative OR Therapy, Alternative OR Non-pharmacological therapy OR Nonpharmacological therapy OR Non-pharmacological therapy OR Nonpharmacological therapy OR Acupuncture* OR Acupuncture OR Massage* OR massage OR acupoint OR Traditional Chinese Medicine Utilitarian OR utilitarian OR Taiji* OR Taiji OR "Tai Chi" OR "Tai-Ji" OR "Tai-Chi" OR Qigong* OR Qigong OR Wuqinxi OR Tendon Changing Classic OR Yijinjing)

5# 3# AND 4#

6# TS=（Randomized controlled trial* OR controlled clinical trial* OR randomized* OR placebo* OR randomly* OR trial* OR groups* OR meta analysis* OR cochrane review* OR systematic review*）

7# 5# AND 6#

**Embase**

#1 ('Miocardial infarction' or 'Angina, Unstable')/exp OR (('Miocardial infarction' OR 'Miocardial infarction' OR 'Disease*, Infarction, Myocardial' OR 'Infarction, Myocardial' OR 'Myocardial Infarctions' OR 'Cardiovascular Stroke' OR 'Cardiovascular Strokes' OR 'Stroke, Cardiovascular' OR 'Myocardial Infarct' OR 'Infarct, Myocardial' OR 'Angina, Unstable' OR 'Anginas, Unstable' OR 'Unstable Anginas' OR 'Angina Pectoris, Unstable' OR 'Angina Pectori, Unstable' OR 'Unstable Angina Pectori' OR 'Unstable Angina' OR 'Angina at Rest' OR 'Angina, Preinfarction' OR 'Preinfarction Angina' OR 'Preinfarction Anginas' OR 'Myocardial Preinfarction Syndrome' OR 'Myocardial Preinfarction Syndromes' OR 'Preinfarction Syndrome, Myocardial' OR 'Syndrome, Myocardial Preinfarction' OR 'Chronic Stable Angina' OR 'Angina Pectoris, Stable' OR 'Stable angina' OR 'Syndromes, Myocardial Preinfarction' OR 'percutaneous coronary intervention' OR PCI OR 'coronary artery bypass grafting'):ti,ab,kw)

#2 'anxiety'/exp OR 'performance anxiety'/exp OR anxiety:ti,ab,kw OR 'social anxiet*':ti,ab,kw OR 'anxiet*, social':ti,ab,kw OR hypervigilance:ti,ab,kw OR nervousness:ti,ab,kw OR anxiousness:ti,ab,kw OR 'performance anxiety':ti,ab,kw OR 'anxieties, performance':ti,ab,kw OR 'anxiety, performance':ti,ab,kw OR 'performance anxieties':ti,ab,kw OR 'panic'/exp OR 'panic':ti,ab,kw OR 'obsessive compulsive disorder'/exp OR 'obsessive compulsive disorder':ti,ab,kw OR 'ocd' OR 'generalized anxiety disorder'/exp OR 'generalized anxiety disorder':ti,ab,kw OR 'gad'/exp OR 'gad':ti,ab,kw OR 'agoraphobia'/exp OR 'agoraphobia':ti,ab,kw OR 'phobic disorders'/exp OR 'phobic disorders':ti,ab,kw OR 'stress disorders':ti,ab,kw OR 'post-traumatic stress disorder'/exp OR 'post

-

traumatic stress disorder' O

R 'ptsd'/exp OR 'ptsd'

#3 #1 AND #2

#4 'complementary therapies'/exp OR 'pharmacological therapy'/exp OR 'nonpharmacological therapy' OR 'complementary therapies':ti,ab,kw OR complementary:ti,ab,kw OR 'therapies, complementary':ti,ab,kw OR 'herapy, complementary':ti,ab,kw OR 'alternative medicine':ti,ab,kw OR 'therapies, alternative':ti,ab,kw OR 'therapy, alternative':ti,ab,kw OR 'non-pharmacological therapy':ti,ab,kw OR 'nonpharmacological therapy':ti,ab,kw

#5 'acupuncture'/exp OR 'massage'/exp OR taiji OR 'qigong'/exp OR acupuncture*:ti,ab,kw OR acupuncture:ti,ab,kw OR massage*:ti,ab,kw OR massage:ti,ab,kw OR acupoint:ti,ab,kw OR 'traditional chinese medicine utilitarian':ti,ab,kw OR utilitarian:ti,ab,kw OR taiji:ti,ab,kw OR 'tai chi':ti,ab,kw OR 'tai-ji':ti,ab,kw OR 'tai-chi':ti,ab,kw OR qigong:ti,ab,kw OR wuqinxi:ti,ab,kw OR 'tendon changing classic':ti,ab,kw OR yijinjing:ti,ab,kw

#6 #4 OR #5

#7 'randomized controlled trial':ti,ab,kw OR 'controlled clinical trial':ti,ab,kw OR randomized:ti,ab,kw OR placebo:ti,ab,kw OR randomly:ti,ab,kw OR trial:ti,ab,kw OR groups:ti,ab,kw OR 'meta analysis':ti,ab,kw OR 'cochrane review':ti,ab,kw OR 'systematic review':ti,ab,kw

#8 #3 AND #6 AND #7

**CNKI search strategy:**

(SU=心肌梗死 OR SU=心梗 OR SU=急性心梗 OR SU=急性冠脉综合征OR SU=不稳定性心绞痛 OR SU=心肌缺血OR SU=PCI) AND (SU=焦虑 OR SU=焦虑表现 OR SU=焦虑症 OR SU=广泛性焦虑 OR SU=紧张焦虑) AND (SU=非药物疗法OR SU=替代疗法 OR SU=针灸 OR SU=按摩OR SU=针刺OR SU=推拿OR SU=穴位 OR SU=灸OR SU=功法OR SU=气功OR SU=太极OR SU=五禽戏OR SU=易筋经OR SU=音乐OR SU=六字诀OR SU=呼吸OR SU=导引) AND (SU=随机 OR FT=随机)

**WanFang search strategy:**

主题:(心梗+心肌梗死+急性冠脉综合征+急性心梗+不稳定性心绞痛+心肌缺血)*主题:(焦虑+焦虑症+焦虑表现+紧张焦虑+广泛性焦虑)*主题:(非药物疗法+替代疗法+针灸+针刺+按摩+推拿+穴位+灸+功法+太极+气功+五禽戏+易筋经+音乐+六字诀+呼吸+导引)*随机

**Chinese scientific journals database search strategy**:

M=(心梗+心肌梗死+急性冠脉综合征+急性心梗+不稳定性心绞痛+心肌缺血)*M=(焦虑+焦虑症+焦虑表现+紧张焦虑+广泛性焦虑)*M=(非药物疗法+替代疗法+针灸+针刺+按摩+推拿+穴位+灸+功法+太极+气功+五禽戏+易筋经+音乐+六字诀+呼吸+导引)*U=随机

**Chinese biomedical literature database search strategy:**

#1 心肌梗死 OR 心梗 OR 急性冠脉综合征OR 不稳定性心绞痛OR PCI

#2 主题词=心肌梗死/全部副主题词

#3 主题词=急性冠脉综合征/全部副主题词

#4 主题词=PCI/全部副主题词

#5 #1~#4/OR

#6 焦虑 OR 焦虑症 OR 紧张焦虑 OR 焦虑样表现 OR 广泛性焦虑

#7 主题词=焦虑/全部副主题词

#8 主题词=焦虑样表现/全部副主题词

#9 主题词=广泛性焦虑/全部副主题词

#10 #6~#9/OR

#11 非药物疗法OR替代疗法OR针灸OR针刺OR按摩OR推拿OR穴位OR灸OR功法OR太极OR气功OR五禽戏OR易筋经OR音乐OR六字诀OR呼吸OR导引

#12 主题词=非药物疗法/全部副主题词

#13 主题词=替代疗法/全部副主题词

#14 主题词=针灸全部副主题词

#15 主题词=推拿/全部副主题词

#17 主题词=穴位贴敷/全部副主题词

#18 主题词=功法/全部副主题词

#19 主题词=太极/全部副主题词

#20 主题词=气功/全部副主题词

#21 主题词=导引/全部副主题词

#22 #11~#21/OR

#23 随机 OR 对照 OR 安慰剂

#24 主题词=随机对照试验[文献类型]

#25 主题词=荟萃分析[文献类型]

#26 主题词=随机分配

#27 主题词=随机对照试验/全部副主题词

#28 #13~#27/OR

#29 #5 AND #10 AND #16 AND #21
