# Supplementary material for: Efficacy and safety of non-pharmacological therapy under the guidance of TCM theory in the treatment of anxiety in patients with myocardial infarction: A protocol for systematic review and meta-analysis
Source: PLoS One. 2023 Jul 6;18(7):e0288154. doi: 10.1371/journal.pone.0288154 (PMC10325115; doi:10.1371/journal.pone.0288154)
Supplement: Attachment — Submitted filename: Response to Reviewers.docx [file pone.0288154.s003.docx]

Title: Efficacy and safety of non-pharmacological therapy under the guidance of TCM theory in the treatment of anxiety in patients with myocardial infarction: a protocol for systematic review and meta-analysis

Dear editor,

The comments of reviewers were extremely insightful and enabled us to greatly improve the quality of our manuscript. In the following pages are our point-by-point responses to each of the comments of the reviewers.We revised the manuscript with red color and made thorough revisions to the article in accordance with reviewers’ suggestions.

We have made numerous corrections to the erroneous sentences and grammar in the original manuscript and have adjusted the article format and file name according to the style requirements of PLOS ONE. Since we have polished the language throughout the entire text, the overall structure of the sentences in “Revised Manuscript with Track Changes” has been adjusted. To make the content changes we made based on the reviewer's opinions more clear, we have marked pagexx and linexx for these modifications in the text following this reply (corresponding to the page and line in “Manuscript clean”).

We hope that with these revisions and our accompanying responses, our manuscript is now suitable for publication in ***PLOS ONE*.**

We shall look forward to hearing from you at your earliest convenience.

Yours sincerely,

Weizhe Zhao

E-mail: zhaoweizhe@126.com

**To Additional Editor**

1. The manuscript requires extensive edits for language, grammar and writing style.

**Response**：Thank you very much for your editing suggestions. We have made extensive revisions and refinements to the language, grammar, and writing style.

 2. "... biggest killer worldwide" - please use more formal and scientific language.

**Response**：I apologize for the grammatical and lexical errors in my previous statement. Here is the revised sentence: " According to an epidemiological report released by the World Health Organization (WHO) in December 2020, ischemic heart disease remains the leading cause of death globally, accounting for 16% of total deaths worldwide" Thank you for your suggestion. In page 3, line 51.

 3. The authors seem to use CHD and myocardial infarction interchangeably, please know that the two are not the same thing, one is a chronic disease and the other is an acute event.

**Response**：I'm sorry for some ambiguity in the words and content I wrote before. We have now unified the research object as patients with myocardial infarction.

 4. In the introduction, you should provide some reference to previous works and statistics on the prevalence of anxiety after cardiac arrest (citation: pubmed.ncbi.nlm.nih.gov/34826580).

**Response**：Thank you for your suggestion. We have now included statistical data on the prevalence of anxiety in patients with myocardial infarction in the article:

Studies have shown that the probability of depression and anxiety after myocardial infarction is about three times that of the general population. Over 50% of patients experience anxiety symptoms within a week after acute myocardial infarction. In page 3, line 56-58.

Data sources：

Lissåker CT, Norlund F, Wallert J, Held C, Olsson EM. Persistent emotional distress after a first-time myocardial infarction and its association to late cardiovascular and non-cardiovascular mortality. Eur J Prev Cardiol. 2019 Sep;26(14):1510-1518.

Wang J, Li P, Qin T, Sun D, Zhao X, Zhang B. Protective effect of epigallocatechin-3-gallate against neuroinflammation and anxiety-like behavior in a rat model of myocardial infarction. Brain Behav. 2020 Jun;10(6):e01633. doi: 10.1002/brb3.1633. Epub 2020 Apr 18.

 5. Were search terms in Mandarin used for the Chinese databases? Please specify.

**Response**：Thank you for your question, the search terms we used when searching the Chinese databases were in Chinese, and the detailed search terms have been elaborated in the search strategy (the search terms can be found in Supporting Information S2_ Search strategy)

6. How would the studies using different psychiatric scales e.g. HADS-A, HAMA, SAS, GAD-7, and DASS-A, be pooled?

**Response**：Thank you very much for your comments. For the different psychiatric rating scales, we will first conduct tests of heterogeneity, if I2 was greater than 50%, indicating the presence of statistical heterogeneity, a subgroup analysis or sensitivity analysis was conducted to eliminate the heterogeneity based on potential heterogeneity factors. If statistical heterogeneity persisted, but clinical homogeneity existed, a random-effects model was employed for conducting meta-analyses. In parallel, we also performed subgroup analyses for different psychiatric scales and applied sensitivity analyses to examine outcome stability. In page 9-10, “Data Analysis”, “Subgroup analysis”.

 **7** "Concomitant with the rising social work pressure in people and the accelerated formation of an aging society" - I am not sure what is this supposed to mean. Please rephrase.

**Response**：Sorry for these miswords and grammatical errors, the word description has now been revised. “With the increasing pressures of modern life and work, combined with a growing elderly population, the incidence of comorbid anxiety and myocardial infarction (MI) is on the rise.” In page 12, line 240-242.

 8. What exactly is "TCM theory"? This is mentioned a few times but authors did not go into any detail about TCM theory.

**Response**：Thank you very much for your suggestion that the elucidation of TCM theory was indeed missing in previous articles now we have included in the article a detailed introduction of TCM theory:

Traditional Chinese Medicine (TCM) provides an explanation for the intricate relationship between myocardial infarction (MI) and emotional disorders from the perspective of Qi, blood, and meridians. TCM theory posits that the unobstructed flow of Qi and blood in the meridians is the foundation of life activities. Pathological conditions such as "blood stasis" and "Qi stagnation" obstructing the meridians can lead to various physical and mental illnesses. Among them, "blood stasis" is the primary pathological product of MI, which can cause obstruction of the heart meridian resulting in symptoms of chest pain and tightness, and can also affect the normal flow of Qi in the meridians, leading to abnormal emotions such as anxiety. To address this complex situation, TCM employs non-pharmacological therapies that differ from herbal treatment. These therapies can enhance psychological and emotional well-being by "regulating Qi and blood circulation in the meridians" while minimizing adverse reactions. In page 4-5, line 85-95.

**Reviewer #1:**

1. The title is slightly confusing and it is unclear if it is related to non pharmacological therapies or TCM.
It will benefit from amendment to reflect non-pharmacological TCM usage.

**Response**：I apologize for the lack of clarity in my previous explanation. In fact, traditional Chinese medicine treatment includes two categories: Chinese herbal medicine treatment and non-pharmaceutical physical therapy. The article mainly focuses on the latter. The concept and detailed introduction of non-pharmaceutical therapy in traditional Chinese medicine have been added to the article. In page 5, line 96-104.

Abstract
2. It may be better to change increase in people's life to "increase in people's life expectancy"

**Response**：Thanks for your help. We have deleted and corrected inappropriate sentences and words in the article.

3. The background section is confusing as it makes its less clear if the authors are inspecting patients with myocardial infarction or CHD

**Response**：Thank you very much for your valuable comments and constructive suggestions. Sorry, the description of included patients in the article may not be appropriate. The words used to describe included patients in the full text are now unified as patients with myocardial infarction.

4. Abstract - A brief sentence on why drug therapy for myocardial infarction and anxiety is controversial.

**Response**：Thanks for your help. Defects in drug treatment have been included in the abstract. As follows: The clinical use of selective serotonin reuptake inhibitors (SSRIs), which are commonly employed, in combination with antiplatelet drugs such as aspirin and clopidogrel, may increase the risk of bleeding in these patients. In page 1-2, line 21-23.

5. Abstract - The authors may wish to elaborate if they are focusing on anxiety symptoms or anxiety disorders or BOTH.

**Response**：Thank you very much for the valuable comments and constructive suggestion. This article focuses on anxiety symptoms. Relevant narratives have been sorted out in the full text to reduce misunderstandings.

6. Abstract - "Utilitarians" may be better worded as "practitioners"

**Response**：We apologize for such inappropriate words errors and the language of the full text has been polished.

7. Abstract - The methods does not describe any inclusion or exclusion criteria for this study in particular the PICO.

**Response**：Thank you for your great suggestion. Your comments are great importance to our article. The PICO of this study has been added to the abstract. As follows: To be eligible for inclusion, studies must diagnose patients with both myocardial infarction and anxiety, and patients must have received non-pharmacological Traditional Chinese Medicine (TCM) therapies such as acupuncture, massage, or Qigong, while the control group received standard treatments. The primary outcome measure will be changes in anxiety scores assessed by anxiety scales. In page2, line 34-38.

Introduction

8. I think it is important to discern if the authors are looking at as they are managed and treated differently.

**Response**：All the included patients will receive non-pharmacological under the guidance of TCM theory intervention. Their specific treatment may be different from acupuncture and moxibustion and massage, but they all belong to the category of non-pharmacological under the guidance of TCM theory intervention. We will use subgroup analysis to explore the source of heterogeneity for their specific treatment.

9. Likewise, discerning whether you are evaluating coronary heart disease patients / patients with recent myocardial infarction is important-> This is due to the title only reflecting patients with myocardial infarction.

**Response**：Thank you very much for your valuable comments and constructive suggestions. Sorry, the description of included patients in the article may not be appropriate. The words used to describe included patients in the full text are now unified as patients with myocardial infarction.

10. "In China, a considerable number of people have a certain resistance to psychotropic drugs, and they are more willing to choose some treatment methods with fewer side effects and higher safety" requires a citation

**Response**：Thank you for your suggestion. We have added the relevant references to the article accordingly. In page4, line 79.

11. The authors describe increased bleeding risk with SSRIs. However, what is the risk and has any recommendation been made by guidelines that SSRI should not be used?

- Clarification needs to be made as to which drugs are controversial to heart disease and its implications

**Response**：It is true that the guidelines do not explicitly state that SSRIs should not be used, but the application of SSRIs to patients with myocardial infarction and anxiety symptoms does have certain limitations. A specific description of bleeding risks has been added to the text. As follows: However, studies have revealed that selective serotonin reuptake inhibitors (SSRIs), a representative class of psychotropic medications, such as sertraline, fluoxetine, and fluvoxamine, carry a heightened risk of bleeding when co-administered with the most frequently used antiplatelet agents following a heart attack, including aspirin or clopidogrel, which can cause bleeding events such as gastrointestinal bleeding and hemorrhagic stroke. In page 4, line 71-76.

12. The authors write initially that the effect of psychotherapy on patients with anxiety and MI is unclear. They latter quote it is not satisfactory.-> There appears to be some contradictions here.

**Response**：Thank you very much for the valuable comments and constructive suggestion. We apologize for such inappropriate words errors and the language of the full text has been polished. As follows : However, studies have revealed that the current psychological therapies have limited efficacy in patients with heart disease and comorbid anxiety. In page 4, line 80-81.

13. Some definition needs to be made with regards to what TCM theory means and what non-pharmacological therapy the authors are looking at?

**Response**：The understanding of traditional Chinese medicine theory on myocardial infarction and anxiety and the concept of non-pharmacological therapy under the guidance of TCM theory will be explained in detail. In page 5, line 96-104.

14. The last paragraph in the introduction lacks citations

**Response**：References have been added to the last paragraph of the introduction. Such as:

Liu H, He Y, Wang J, Miao J, Zheng H, Zeng Q. Epidemiology of depression at Traditional Chinese Medicine Hospital in Shanghai, China. Compr Psychiatry. 2016 Feb;65:1-8. doi: 10.1016/j.comppsych.2015.10.004

Amorim D, Amado J, Brito I, Fiuza SM, Amorim N, Costeira C, Machado J. Acupuncture and electroacupuncture for anxiety disorders: A systematic review of the clinical research. Complement Ther Clin Pract. 2018 May;31:31-37. doi: 10.1016/j.ctcp.2018.01.008

15. Given the heterogeneity of data collected, I am uncertain if a meta-analysis may be most appropriate for TCM trials
-> Perhaps a scoping review may be more appropriate.

**Response**：Meta-analysis can be applied to Traditional Chinese Medicine (TCM) trials, as it is a systematic method for integrating and analyzing the results of multiple independent studies. TCM trials can be conducted using rigorous scientific methods for experimental design, data collection, and statistical analysis, thus producing comparable and statistically meaningful results. Consequently, meta-analysis can be used to integrate and statistically analyze the results of multiple TCM trials, yielding more reliable and statistically significant conclusions.

We will screen and assess each study, excluding those of poor quality or non-standardized protocols, while ensuring the comparability of the data sources and study populations.

For example：

Zhang J, Zhang Z, Huang S, Qiu X, Lao L, Huang Y, Zhang ZJ. Acupuncture for cancer-related insomnia: A systematic review and meta-analysis. Phytomedicine. 2022 Jul 20;102:154160.

Ji W, Wu L, Pan G, Zou X. Effects and Safety of Non-Pharmacological Therapies of Traditional Chinese Medicine for Coronary Heart Disease: An Overview of Systematic Reviews. Evid Based Complement Alternat Med. 2022 Mar 19;2022:8465269.

**Methods**

16. Was the PRISMA 2020 guidelines used?

**Response**：We apologize for the omission in the article. This meta-analysis will be carried out under the guidance of PRISMA 2020 guidelines, relevant content has been added to the article. As follow: It will adhere to the Cochrane Handbook and the Preferred Reporting Items for Systematic Reviews and Meta-analysis Protocols (PRISMA-P) guidelines. In page 7, line 148-149.

17. For the anxiety, were a psychiatrist involved in the diagnosis?

-> Noted that the authors mentioned a cut off of >=7 on HADS-A for diagnosis for anxiety

-> However, HADS-A is not used for diagnosis of anxiety disorders and instead valiated for diagnosis of anxity symptoms

**Response**：Thank you very much for the valuable comments and constructive suggestion. The main focus of the study was the symptoms of anxiety. So all patients included in this study have a clinical compliance anxiety scale score of anxiety level (such as HADS-A ≥ 7). The reason we included patients with anxiety disorders is because these patients all have anxiety symptoms. To avoid missing any relevant information, we incorporated studies focusing on patients with anxiety disorders and analyzed the changes in anxiety scales found within their articles. The diagnoses of anxiety disorders in the studies that have been included are explicitly determined by psychiatrists who are part of the research process, rather than by us.

18. What are the exclusion for participants in the study?

**Response**：We apologize for the omission in the article. We have now improved the exclusion criteria in the study participants section of the text. As follow: Exclusion criteria: (1) patients with severe heart failure who are unable to receive the research intervention. (2) patients with a history of mania, consciousness disorders, or other serious mental illnesses. (3) literature that cannot provide detailed outcome indicators. (4) duplicate publications. In page 7 line 130-132.

19. What as the rationale for assessing cardiopulmonary function as an outcome in this study?

**Response**：Assessing cardiopulmonary function can provide a better understanding of the cardiac function status of patients. It is used as one of the outcome measures in the study to investigate the effects of non-pharmacological Traditional Chinese Medicine therapies on cardiac function in patients with acute myocardial infarction and comorbid anxiety.

20. The authors need to be clear on whether they are assessing anxiety disorders / symptoms for the outcomes.

**Response**：Thank you very much for the valuable comments and constructive suggestion. The main focus of the study was the symptoms of anxiety. So all patients included in this study have a clinical compliance anxiety scale score of anxiety level (such as HADS-A ≥ 7), and we will use the changes in anxiety scale scores as the primary outcome measure.

21. Search terms from other anxiety related reviews and traditional chinese medicine should be employed in this review and cited as appropriate

-> https://pubmed.ncbi.nlm.nih.gov/33516963/

-> https://www.ncbi.nlm.nih.gov/pmc/articles/PMC4951626/

-> https://www.cureus.com/articles/127502-role-of-alternative-medical-systems-in-adult-chronic-kidney-disease-patients-a-systematic-review-of-literature

-> https://pubmed.ncbi.nlm.nih.gov/35104758/

**Response**：We greatly appreciate your suggestion. We have already added relevant terms to the main search keywords section. Detailed and specific anxiety-related search terms and Traditional Chinese Medicine non-pharmacological therapy search terms are listed in the search strategy appendix. Additionally, we have cited reference： https://pubmed.ncbi.nlm.nih.gov/35104758

22. Potential limitations of this review should be included

-> e.g. language, limits of evaluating only RCTs

**Response**：Thank you for your suggestion. We have incorporated potential limitations, such as language and types of included literature, into the discussion section:

This study has some potential limitations. In terms of language, we only included studies in Chinese and English, and other studies using non-pharmacological TCM therapies in different languages could not be effectively incorporated. Additionally, this study only evaluated the results of RCTs and did not conduct a comprehensive analysis of other types of studies such as cohort studies and observational studies. This study lays a foundation for future research and provides new evidence-based medicine support for clinical application. Future studies could expand the breadth of research, include studies in different languages and types, fully consider limitations and biases, and conduct more in-depth and reasonable research and discussion. In page12-13.

Language

23. Significant amount of grammatical errors

-> To consider English editing services as appropriate

**Response**：We apologize for the presence of these grammatical errors. We have implemented extensive revisions to correct the grammar and refine the language.

**Reviewer #2:**

1. Authors should define ischemic heart disease for the lay reader and to keep consistency with the fact that the term anxiety is defined within the first paragraph.

**Response**：I'm sorry for the confusion caused by the ambiguous wording and content in our previous writing. We have now clarified that the research will focus on patients with myocardial infarction.

2.Define psychotherapy and psychotropic drugs to provide context for the reader.

**Response**：Thank you very much for your suggestion. We have now added relevant descriptions of psychological therapies and psychotropic medications used for treating anxiety in the article. In page4, line 68-71.

3. For the acronym ‘SSRIs’, write out the acronym in full when it is first introduced.

**Response**：I'm sorry for the mistake. The necessary changes have been made in the article.

4. For discussion of traditional Chinese medicine, define ‘Taiji’ for the lay reader.

**Response**：Thank you very much for your suggestion. We have now provided an explanation of the concept and characteristics of non-pharmaceutical therapies in traditional Chinese medicine, such as Tai Chi and other exercises. In page5, line 96-104.

5. For type of interventions for the control group, the use of the word ‘sham acupuncture’ is curious. Authors should explain this.

**Response**：Thank you very much for the question, "Sham acupuncture" is a commonly used control group in acupuncture clinical trials. It involves the use of a non-penetrating needle, also known as a placebo needle or a blunt needle, that is designed to mimic the sensation of real acupuncture without actually penetrating the skin or stimulating the acupuncture points. The purpose of using a sham acupuncture control group is to help researchers determine the true efficacy of real acupuncture by minimizing the placebo effect and any potential biases.

6. For the search strategy, in “The two evaluators will independently conduct systematic searches on PubMed, Embase, Ovid, Scopus, Cochrane Central Controlled Trial Registration Center, Web of Science, China Knowledge Resource Integration Database and Wanfang Database from their establishment to December 31, 2022”, is December 31, 2022, the end date by which studies have to be published to be eligible? Is there a beginning date for eligibility?

**Response**：I'm sorry for the unclear language in my previous description. The research included articles published up to December 31, 2022. The sentence has been revised accordingly. In page7-8, line 149-153.

7. Capitalize pronouns in ‘2.10 ethics and dissemination’, ‘2.11 patient and public involvement’ and ‘2.12 amendments (amending)’.

**Response**：I'm sorry for the writing errors. The necessary corrections have been made in the text.

8. Discuss anticipated implications of your research.
Overall, this is a relevant topic that can make a unique contribution to the literature. Consider expanding discussion on the background and significance of the study, clearly detailing the steps in the research methodology and describing anticipated implications of this research.

**Response**：Thank you very much for your suggestion! We hope that this research will provide higher-level clinical evidence for the safety and effectiveness of non-pharmaceutical therapies in traditional Chinese medicine for treating anxiety in patients with myocardial infarction, and offer more treatment options for clinical doctors. We have further added a discussion of the background and significance of the research and provided a more detailed explanation of the research plan.
