# Supplementary material for: Efficacy and safety of non-pharmacological therapy under the guidance of TCM theory in the treatment of anxiety in patients with myocardial infarction: A protocol for systematic review and meta-analysis
Source: PLoS One. 2023 Jul 6;18(7):e0288154. doi: 10.1371/journal.pone.0288154 (PMC10325115; doi:10.1371/journal.pone.0288154)
Supplement: Attachment — Submitted filename: Response to Reviewers.docx [file pone.0288154.s004.docx]

Title: Efficacy and safety of non-pharmacological therapy under the guidance of TCM theory in the treatment of anxiety in patients with myocardial infarction: a protocol for systematic review and meta-analysis

Dear editor,

The comments of reviewers were extremely insightful and enabled us to greatly improve the quality of our manuscript. In the following pages are our point-by-point responses to each of the comments of the reviewers. We revised the manuscript with red color and made revisions to the article in accordance with reviewers’ suggestions.

We have meticulously revised and polished the language of the manuscript through a professional English editing service. Certificate of this refinement can be seen in “S3_Certificate of editing”. To make the content changes we made based on the reviewer's opinions more clear, we have marked pagexx and linexx for these modifications in the text following this reply (corresponding to the page and line in “Manuscript”).

We hope that with these revisions and our accompanying responses, our manuscript is now suitable for publication in ***PLOS ONE*.**

We shall look forward to hearing from you at your earliest convenience.

Yours sincerely,

Weizhe Zhao

E-mail: zhaoweizhe@126.com

**To Additional Editor**

1. close edits for language still necessary throughout the manuscript

**Response：**Thank you for your suggestions. We have meticulously revised and polished the language of the manuscript through a professional English editing service. Certificate of this refinement can be seen in “S3_Certificate of editing”. We appreciate your valuable input in helping us improve our work.

2. in the introduction, you should provide some reference to previous works and statistics on the prevalence of anxiety after cardiac arrest (citation: pubmed.ncbi.nlm.nih.gov/34826580). Ref [5] is not specific for anxiety and a meta-analysis would provide a better estimate than a single study.

**Response：**Thank you for your valuable suggestion. We have incorporated the relevant data on the prevalence of anxiety following cardiac arrest (pubmed.ncbi.nlm.nih.gov/34826580) into our manuscript, as per your recommendation. In page3, line 57-59. This addition will indeed enhance the accuracy and comprehensiveness of our description.

3. how would the studies using different psychiatric scales e.g. HADS-A, HAMA, SAS, GAD-7, and DASS-A, be pooled?

**Response：**Thank you for raising this question. When evaluating the same outcome using different methods, such as various psychiatric scales (HADS-A, HAMA, SAS, GAD-7, and DASS-A) for measurement, we employ the Standardized Mean Difference (SMD). The SMD allows us to account for variability between different scales by standardizing the results, enabling us to compare and combine the outcomes from various instruments. In page10, line 204-208.

4. how does the authors intend to handle the high study heterogeneity in the meta-analysis? Would provisions be made for a systematic review without meta-analysis?

**Response：**Thank you for raising this valuable concern. We have now included the methods for addressing high heterogeneity among the studies in our meta-analysis within the manuscript：In the event of high heterogeneity during the meta-analysis process, several steps will be taken: 1) We will perform a subgroup analysis, categorizing studies based on potential sources of heterogeneity, followed by separate meta-analyses for each subgroup. 2) Meta-regression methods will be used to analyze study characteristics and identify potential features contributing to heterogeneity. 3) If the issue of high heterogeneity cannot be resolved effectively, we will consider converting the meta-analysis into a systematic review, thereby offering a qualitative synthesis of studies without quantitative aggregation. In page11, line 216-222.

**To Reviewer 1**

Dear Reviewer,

Thank you for taking the time to review our manuscript and providing your constructive feedback. We sincerely appreciate your valuable comments and suggestions. In accordance with your feedback, we have revised the manuscript and subjected the language to rigorous editing and refinement through a professional English editing service (S3_Certificate of editing). The specific modifications to the content of the article are as follows:

1.-> It is generally well accepted that significant heterogenity exists for TCM related trials
--> How do the authors plan to account for the methodological, clinical heterogenity of included studies?

**Response：**Thank you for your critical questions. We acknowledge that trials related to traditional Chinese medicine often exhibit significant heterogeneity, which is a factor we must consider in conducting a meta-analysis. To address the methodological and clinical heterogeneity in the included studies, we propose the following measures:**1.Methodological heterogeneity resolution:** In selecting studies, we will adhere to rigorous quality assessment criteria, incorporating only randomized controlled trials (RCTs) to ensure consistency in our research design and implementation. Furthermore, we will use risk of bias tools from networks such as the Cochrane Collaboration to evaluate the quality of each study, thereby controlling the potential impact of study quality. **2.Clinical heterogeneity resolution:** We will standardize the criteria for including studies and strictly adhere to these criteria. During the data collection phase, we will meticulously record various clinical information, such as the treatment methods received by patients and the employed assessment scales. While the specific interventions that patients receive may vary, all these therapeutic methods are rooted in the theories of Qi and blood, along with the meridian system as articulated in Traditional Chinese Medicine. Hence, they share a unifying core treatment philosophy. Beyond this, we will employ subgroup analysis and meta-regression to explore and explain these clinical heterogeneities. For instance, we will conduct further subgroup analyses based on different types of treatment methods. Besides, we will conduct a sensitivity analysis to test the robustness of our results. If the removal of a particular study leads to significant changes in the outcome, we will delve into a thorough discussion of the implications. From page10, line 199 to page12, line 243.( Data Analysis; Subgroup analysis; Sensitivity Analysis; Grading the Quality of Evidence)

2.1The authors informed that they included HADS-A to screen for anxiety symptoms among patients with anxiety disorders
-> While anxiety symptoms can be screened by HADS-A, it is more used as a screening tool for anxiety symptoms in the general population than patients with anxiety. Further clarification needs to be made.
-> If HADS-A is used, what is defined as the Minimally clincially important difference?

**Response：**Thank you for highlighting the need for further clarification regarding the use of HADS-A to screen for anxiety symptoms in our study. We appreciate your expertise in this area and acknowledge that HADS-A is more commonly used as a screening tool for anxiety symptoms in the general population. The purpose of listing HADS-A in the manuscript was to provide an example for " Patients with a clinical compliance anxiety scale score of anxiety level " rather than to evaluate all patients with anxiety symptoms. As some of the included studies involve patients with anxiety disorders, they often employ the HAMA scale for assessment. To avoid ambiguity, we have further clarified and revised this part of the manuscript. It is worth noting that our research primarily focuses on the changes in anxiety symptoms; hence, we included patients with anxiety disorders, as they exhibit these symptoms. To avoid omitting any relevant information, we incorporated studies involving myocardial infarction patients with concurrent anxiety disorders and analyzed changes in anxiety scales reported in their articles.(The diagnoses of anxiety disorders in the included studies were determined by psychiatrists involved in the research process) For evaluating different scales, we use the SMD to account for the variability between them by standardizing the results, enabling us to integrate and compare their outcomes. In page6, line 124-128.

2.2-> If HADS-A is used, what is defined as the Minimally clincially important difference?
-> How is the cutoff of 7 decided and not 8 used for other studies?

**Response：**We sincerely apologize for the oversight in our manuscript. It was an unintended error that we mistakenly wrote HADS-A ≥ 7 instead of HADS-A > 7. We understand that this may have caused confusion in your interpretation of our findings. We have now corrected this error in the manuscript to accurately reflect "HADS-A > 7." In page6, line 125. Thank you for your understanding.

3.Clarification needs to be made about what consciousness disorders and other serious mental illness entails.

**Response：**Thank you for your suggestion. We have further clarified the consequences of altered consciousness and other severe mental illnesses in the manuscript: Patients with a history of mania, consciousness disorders, or other serious mental illnesses include schizophrenia, bipolar disorder, and severe major depressive disorder. Patients with consciousness disorders may be unable to accurately communicate for scale evaluations, and other severe mental illnesses could potentially influence participants' responses to treatment or interfere with anxiety symptoms, thereby affecting research outcomes. Furthermore, the efficacy of traditional Chinese medicine on this patient population remains unclear. Excluding them helps mitigate the risk of exacerbating their conditions. In page7, line 132-137.

4.Please make the necessary citation as they contain important search terms relevant to your article and will add to the robustness of your review

-> https://pubmed.ncbi.nlm.nih.gov/33516963/
-> https://www.ncbi.nlm.nih.gov/pmc/articles/PMC4951626/
-> https://pubmed.ncbi.nlm.nih.gov/28064110/
-> https://www.cureus.com/articles/127502-role-of-alternative-medical-systems-inadult-chronic-kidney-disease-patients-a-systematic-review-of-literature
-> https://pubmed.ncbi.nlm.nih.gov/35104758/

**Response：**Thank you for your valuable suggestions and guidance, including the recommendation to incorporate additional references. We have carefully reviewed the suggested articles and have included the following references in our manuscript to enhance the robustness of our review.

https://pubmed.ncbi.nlm.nih.gov/33516963/
https://www.ncbi.nlm.nih.gov/pmc/articles/PMC4951626/

<https://www.cureus.com/articles/127502-role-of-alternative-medical-systems-inadult-chronic-kidney-disease-patients-a-systematic-review-of-literature>

(In page8, line 161-162, as well as Table 1.and other databases in the “S_2 Search strategy”)

https://pubmed.ncbi.nlm.nih.gov/35104758/ (In page13, line 275-276.)

Once again, thank you for your invaluable feedback.

**To Reviewer 2**

Dear Reviewer,

We greatly appreciate your positive feedback on the revised manuscript and are pleased to know that our efforts to improve clarity and provide more detailed explanations have met your expectations. Now we have meticulously revised and polished the language of the manuscript through a professional English editing service. We are grateful for your guidance and support throughout the revision process, which has contributed to enhancing the quality of our work. Thank you for considering our manuscript suitable for publication.
