# Supplementary material for: Efficacy and safety of non-pharmacological therapy under the guidance of TCM theory in the treatment of anxiety in patients with myocardial infarction: A protocol for systematic review and meta-analysis
Source: PLoS One. 2023 Jul 6;18(7):e0288154. doi: 10.1371/journal.pone.0288154 (PMC10325115; doi:10.1371/journal.pone.0288154)
Supplement: Attachment — Submitted filename: Response to Reviewers.docx [file pone.0288154.s005.docx]

**Title: Efficacy and safety of non-pharmacological therapy under the guidance of TCM theory in the treatment of anxiety in patients with myocardial infarction: a protocol for systematic review and meta-analysis**

Dear editor,

Thank you very much for your feedback on the manuscript. Your comments were extremely insightful and enabled us to greatly improve the quality of our manuscript. In the following pages are our point-by-point responses to each of the comments. We revised the manuscript with red color and made revisions to the article in accordance with your suggestions.

We fully understand your concerns about the varying quality of evidence supporting Traditional Chinese Medicine's pathophysiology and therapies, and we fully agree that caution should be exercised when discussing this topic. Following your advice, we have made revisions to the descriptions in the manuscript. concerning this aspect, clearly indicating that they just belong to the realm of theoretical or hypothetical propositions within Traditional Chinese Medicine, rather than established facts. To make the content changes we made based on the your opinions more clear, we have marked pagexx and linexx for these modifications in the text following this reply (corresponding to the page and line in “Manuscript”).

We hope that with these revisions and our accompanying responses, our manuscript is now suitable for publication in ***PLOS ONE*.**

We shall look forward to hearing from you at your earliest convenience.

Yours sincerely,

Weizhe Zhao

E-mail: zhaoweizhe@126.com

**To Editor：**

Because of the varying quality of evidence supporting TCM pathologies and therapies, care should be taken to avoid statements indicating their effectiveness or that encouraging the use of TCM diagnoses in medical care. Theories of TCM can be discussed provided they are framed as such. However, the manuscript should not include statements indicating that TCM therapies are effective. The manuscript should not include presentation of TCM theories presented as fact. Specific examples, from lines 86-103 that require revision can be found below:

**Response：**Thank you very much for your feedback on the manuscript. We fully understand your concerns about the varying quality of evidence supporting Traditional Chinese Medicine's pathophysiology and therapies, and we fully agree that caution should be exercised when discussing this topic. We have made revisions to the descriptions in the article concerning this aspect, clearly indicating that they just belong to the realm of theoretical or hypothetical propositions within Traditional Chinese Medicine, rather than established facts. Additionally, we have also modified the statements indicating the effectiveness of TCM treatments to ensure the rigor of the manuscript.

1) "Pathological conditions such as "blood stasis" and "Qi stagnation" obstructing the meridians can lead to various physical and mental illnesses" - should be revised to "TCM theory suggests that pathological conditions such as "blood stasis" and "Qi stagnation" obstructing the meridians can lead to various physical and mental illnesses" or similar. A suitable reference must be provided.

**Response：**Thank you for your valuable suggestion. We have revised the manuscript to underscore that this portion reflects the perspective of Traditional Chinese Medicine, and we have appended relevant references for further substantiation. In page5, line 87-90.

2) A similar edit is required to the sentence ""Blood stasis" is the primary pathological product of MI, which can obstruct the heart meridian resulting in symptoms of chest pain and tightness, it can also affect the normal flow of Qi in the meridians, leading to abnormal emotions such as anxiety". A reference must be required.

**Response：**Thank you for your suggestion. We have revised and edited this section accordingly, and provided the corresponding references. In page5, line 87-90.

3) The following sentence is missing a reference. "These therapies can enhance psychological and emotional well-being by "regulating Qi and blood circulation in the meridians" while minimizing adverse reactions." A reference must be provided and the text updated to indicate that "Evidence has suggested that these therapies..." or similar.

**Response：**We are deeply grateful for your invaluable advice. We have rigorously revised the text to emphasize that “evidence suggests these therapies can improve mental states” and we have provided the corresponding references. In page5, line 94-96.

4) Several other statements from lines 86-103 are missing references. Reference 16 does not appear to support the claim made. Please carefully revise the text in this section to provide suitable references for all statements made

**Response：**We sincerely apologize for the inappropriate citation of Reference [16]. We have now corrected Reference [16] and supplemented the references for this portion, providing more persuasive sources to support the discussion on Traditional Chinese Medicine theory in this paragraph. In page5, line 86-104.

Finally, Please review your reference list to ensure that it is complete and correct. If you have cited papers that have been retracted, please include the rationale for doing so in the manuscript text, or remove these references and replace them with relevant current references. Any changes to the reference list should be mentioned in the rebuttal letter that accompanies your revised manuscript. If you need to cite a retracted article, indicate the article’s retracted status in the References list and also include a citation and full reference for the retraction notice.

**Response：**Thank you very much for your suggestion. We have conducted a further review and verification of our references. We found an error in the formatting of Reference 14, which we have now corrected. We appreciate your assistance once again!

**To Reviewers**

Dear Reviewer,

We greatly appreciate your positive feedback on the revised manuscript and are pleased to know that our efforts to improve clarity and provide more detailed explanations have met your expectations. We are grateful for your guidance and support throughout the revision process, which has contributed to enhancing the quality of our work. Thank you for considering our manuscript suitable for publication.
